# Assessment of Drug Utilization Pattern of Antimicrobials Prescribed in the Orthopedic Department of a Tertiary Care Hospital

**DOI:** 10.7759/cureus.111517

**Published:** 2026-06-25

**Authors:** Kartiki P Patil, Vandana M Thorat, Leesha L Chawla

**Affiliations:** 1 Department of Pharmacology, Krishna Institute of Medical Sciences, Krishna Vishwa Vidyapeeth (Deemed To Be University), Karad, IND

**Keywords:** antimicrobials, cephalosporins, ciprofloxacin, drug utilization, fixed dose combinations, metronidazole, orthopedics, polypharmacy, prescription, road traffic accidents

## Abstract

Introduction

The purpose of prescription patterns is to collect valid data, which would help in formulating appropriate guidelines to ensure the rational use of medicines. Unfortunately, information about the use of medicines in patients visiting orthopedic clinics in tertiary care hospitals in India, especially in Maharashtra, is scanty. Therefore, the present study was undertaken with the primary objective to evaluate the current drug-prescribing trend of antimicrobials in the management of inpatients of the orthopedic department and secondary objectives to assess group-wise distribution of antimicrobials prescribed to inpatients of the orthopedic department and to assess additional drugs prescribed to these patients.

Methods

This study was a cross-sectional observational study done on 99 patients who were admitted to the wards of the orthopedics department of the hospital. The drug utilization assessment was done by using the WHO core drug prescribing indicators. The assessment of the drugs prescribed according to the essential drug list was done using the National List of Essential Drugs Medicines (NLEM) 2022 of India.

Results

The study population was predominantly elderly (47.5% ≥60 years) with male predominance (62.6%), and falls (58.6%) as the leading cause, followed by road traffic accidents (35.3%). Antimicrobial use was dominated by broad-spectrum agents, particularly ceftriaxone (43.4%), ciprofloxacin (47.5%), and metronidazole (54.5%), with moderate use of aminoglycosides (35.4%). Fixed-dose combinations were led by Cefuroxime + Sulbactam (25.2%). Supportive therapy was extensive, including ranitidine (62.6%), tramadol (59.6%), antihypertensives, insulin, and nutraceuticals. Combination therapies such as Bromelain + Trypsin + Rutoside (37.4%) were frequently used.

Conclusion

This study reflects a pattern of polypharmacy characterized by extensive use of broad-spectrum antimicrobials and adjunctive therapies, emphasizing the need for rational prescribing practices and antimicrobial stewardship to optimize clinical outcomes and minimize resistance.

## Introduction

Drug utilization is described as the promotion, supply, prescription, and administration of drugs by a community, with an emphasis on the consequences of the drugs used medically, socially, and economically [1]. Inappropriate use of medicines contributes to increased morbidity and mortality, imposes additional financial burden, compromises drug quality, and leads to wastage of resources. It also enhances the risk of adverse drug reactions and promotes the development of resistance [2]. Rational use of medicines focuses on evaluating the availability, accessibility, and appropriate prescribing practices. In countries such as India, implementing rational drug use is especially important due to constrained financial resources and limited patient affordability [3].

Infections pose a major challenge in orthopedic practice, largely due to trauma, open fractures, implant-related procedures, and prosthetic joint replacements, all of which elevate the risk of microbial contamination. Orthopedic conditions, including fractures, joint disorders, and postoperative wound complications, frequently necessitate antimicrobial therapy for either prophylactic purposes or treatment of established infections. Consequently, antibiotics are among the most often preferred drugs in orthopedic departments to prevent surgical site infections and manage bone and soft tissue infections [4].

Antimicrobials are agents used for the prevention or treatment of infections and encompass antibiotics, antivirals, antifungals, antiparasitics, and antiseptics. The discovery of penicillin by Sir Alexander Fleming in 1928 marked the beginning of the antibiotic era and significantly transformed modern medicine [5]. Orthopedic departments employ a wide range of drugs from multiple classes to meet therapeutic demands. However, this diversity may contribute to drug interactions, adverse drug reactions, and prescribing errors, ultimately increasing healthcare costs and negatively affecting patient adherence [6].

The unnecessary prescription of antibiotics can lead to several complications. While antibiotics are life-saving when used appropriately for bacterial infections, their indiscriminate use contributes to the emergence and spread of antibiotic-resistant organisms. Therefore, judicious reduction in antibiotic use is essential to limit resistance and associated diseases [7]. WHO has identified antimicrobial resistance (AMR) as a major global health threat and one of the most pressing public health challenges [8].

In response, WHO has developed a global action plan outlining five key objectives to combat antibiotic resistance [9], one of which emphasizes the optimization of antimicrobial use [10]. Evaluating drug utilization is essential for clinical, educational, and pharmacoeconomic purposes [1]. Prescription pattern studies provide accurate data that support the development of local guidelines for rational drug use by comparing prescribing practices with established standards [11]. However, there is limited information regarding drug utilization patterns among patients attending orthopedic outpatient departments in public tertiary care hospitals [12]. Inappropriate, insufficient, and economically unjustified use of antibiotics remains a widespread issue in healthcare systems globally, particularly in developing communities [13]. Additionally, in Maharashtra and the broader Indian context, only a few studies have been conducted. By the conclusion of this study, a clearer understanding of antimicrobial utilization patterns in orthopedic practice will be achieved, highlighting the importance of their rational use.

With this context, the present study was conducted with the main purpose of studying the drug utilization pattern of antimicrobials and additional drugs prescribed in the orthopedic department at our tertiary care hospital.

## Materials and methods

Study design and population

The present investigation employed a cross-sectional observational study conducted among 99 patients admitted to the wards of the orthopedic department at Krishna Charitable Hospital & Medical Research Centre, Krishna Institute of Medical Sciences (KIMS), Krishna Vishwa Vidyapeeth (KVV), Karad, Maharashtra. The study was carried out over a period of one and a half years from March 2024 to August 2025. The approval for study conduct was obtained from the Institutional Ethics Committee of Krishna Vishwa Vidyapeeth (Deemed To Be University), Karad (approval number: KVV/IEC/05/2024; protocol No. 352/2023-2024), dated April 18, 2024. The patients were recruited using convenience sampling from patients admitted to the orthopedic ward who were prescribed at least one antimicrobial agent for the management of an orthopedic condition during the study period. Patients fulfilling these criteria were enrolled for prescription review and analysis. As this was a non-interventional, observational study involving only the review and analysis of prescriptions and medical records, no direct patient contact or intervention was undertaken. Therefore, individual informed consent was waived by the Institutional Ethics Committee. Patient confidentiality and anonymity were maintained throughout the study. The study rationale consisted of prescription sheets of the participants, which were evaluated, and all data relevant to the study variables were collected.

Inclusion Criteria

Patients admitted to the orthopedic ward, irrespective of their gender, and those treated with at least one antimicrobial agent.

Exclusion Criteria

Patients taking antimicrobials for conditions other than orthopedic ones, pregnant, or lactating women.

Sample size

The sample size was calculated using the following formula based on the most prevalent use of the antimicrobial agent (48.6% for ceftriaxone) received, reported in a previous study [4]. The sample size was calculated using the formula N = Z^2^pq/L^2^. Here, N denotes the required sample size, Z represents the standard normal deviate corresponding to the desired confidence level (95% confidence interval), p refers to the expected prevalence (48.6%) obtained from previous studies [4], q = 100−p = 51.4%, L denotes the absolute precision of 10%. Substituting the above values in the formula, N = (2)^2^x 0.486 x 0.514 / (0.10)^2^ = 4 x 0.2498/0.01 = 99.92, which was rounded to 99, considering feasibility and the study duration. Therefore, a total of 99 patients were included in the study.

Data collection and assessment parameters

Data were collected using a structured case record form. Prescriptions of patients admitted to the orthopedic ward were reviewed at a single point in time. For each patient, only one prescription was included in the analysis to avoid duplication and repeated inclusion of the same patient. The prescriptions were analyzed in accordance with the study objectives. Basic demographic data of the patients, total number of drugs prescribed to the patients, dose regimen of the drugs prescribed to the patients (i.e., dose, route, and dosage form), and fixed-dose combinations (FDCs) prescribed to the patients were documented. The collected prescription data were analyzed using descriptive statistics. Drug utilization patterns were expressed in terms of frequencies, percentages, and proportions. All relevant study parameters were summarized as percentages. The drug utilization pattern was evaluated using the WHO core drug prescription indicators. Drugs prescribed from the list of essential drugs were evaluated using the National List of Essential Medicines (NLEM), 2022, of India.

Statistical analysis

Data were entered in MS Excel, 2021 (Microsoft Corporation, Redmond, Washington), and analysis was done using IBM SPSS Statistics for Windows, Version 27 (Released 2020; IBM Corp., Armonk, New York). Data were analyzed using descriptive statistics. Categorical variables were represented in the form of percentages and frequencies.

## Results

Sociodemographic characteristics

Table 1 describes the sociodemographic characteristics of the study population. The study population was predominantly elderly, with 47 (47.5%) aged ≥60 years, indicating a higher burden in the geriatric group. Males constituted the majority, 62 (62.6%), suggesting greater exposure to risk factors. Falls were the leading cause, 58 (58.6%), followed by road traffic accidents, 35 (35.3%), while post-operative complications were minimal, 6 (6.1%). Overall, the findings highlight age-related vulnerability and trauma, particularly falls as the primary contributing factors.

**Table 1:** Socio-demographic and patient characteristics. Data have been represented as N (%) unless otherwise stated. RTA: road traffic accidents.

Variables	N (%)
Age (years)	
<20	3 (3.0)
21-39	29 (29.3)
40-59	20 (20.2)
≥60	47 (47.5)
Gender	
Male	62 (62.6)
Female	37 (37.4)
Causes	
RTAs	35 (35.3)
h/o fall	58 (58.6)
Post-operative complications	6 (6.1)

Antimicrobial drug classes

Table 2 describes the antimicrobial class of drugs prescribed in the orthopedic department. Cephalosporins constituted the most frequently utilized antimicrobial class, with ceftriaxone being the predominant agent, 43 (43.4%), followed by cefuroxime, 9 (9.1%), and cefotaxime, 2 (2.0%), reflecting a preference for broad-spectrum β-lactam therapy. Among quinolones, ciprofloxacin showed high utilization, 47 (47.5%), indicating its significant role in empirical treatment. Aminoglycosides demonstrated moderate usage, with tobramycin at 18 (18.2%) and gentamicin at 17 (17.2%), often suggestive of combination therapy in severe infections. Notably, metronidazole showed the highest individual drug utilization, 54 (54.5%), emphasizing the importance of anaerobic coverage. Other antimicrobial classes, including macrolides - clarithromycin, 1 (1.0%); tetracyclines - tetracycline, doxycycline, and tigecycline, each 1 (1.0%); oxazolidinones - linezolid, 1 (1.0%); and anthelmintics - mebendazole and diethylcarbamazine, 5 (5.0%) each, were minimally prescribed. Overall, the prescribing pattern indicates a strong reliance on broad-spectrum antibiotics, particularly cephalosporins, quinolones, and nitroimidazoles, with limited use of other antimicrobial classes.

**Table 2:** Antimicrobial class of drugs prescribed in orthopedic department. Data have been represented as numbers (percentage).

Antimicrobial class of drugs	Drugs	N (%)	Cumulative (%)
Cephalosporins	Cefotaxime	2 (2.0)	54.5
	Cefuroxime	9 (9.1)	
	Ceftriaxone	43 (43.4)	
Quinolones	Ciprofloxacin	47 (47.5)	47.5
Aminoglycosides	Gentamicin	17 (17.2)	35.4
	Tobramycin	18 (18.2)	
Macrolides	Clarithromycin	1 (1.0)	1.0
Tetracyclines	Tetracycline	1 (1.0)	3.0
	Doxycycline	1 (1.0)	
	Tigecycline	1 (1.0)	
Nitroimidazole	Metronidazole	54 (54.5)	54.5
Anthelmintics	Mebendazole	5 (5.0)	10.0
	Diethylcarbamazine	5 (5.0)	
Oxazolidinone	Linezolid	1 (1.0)	1.0

FDC prescriptions

**Figure 1:**
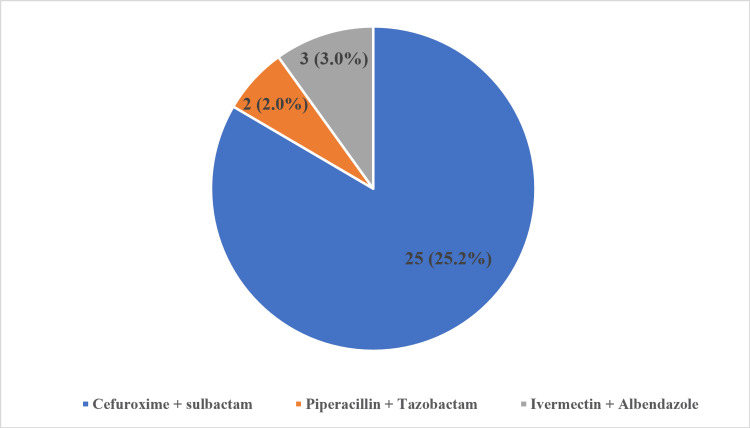
Fixed-dose combinations (FDCs) prescribed in the orthopedic department. Data have been represented as numbers (percentage).

Figure 1 depicts the FDCs prescribed in the orthopedic department. The FDC therapy was predominantly represented by cefuroxime + sulbactam, accounting for 25 (25.2%) of prescriptions, indicating a preference for β-lactam/β-lactamase inhibitor combinations to enhance antimicrobial coverage. In contrast, piperacillin + tazobactam was minimally utilized 2 (2.0%), suggesting limited use of higher-end broad-spectrum combinations. The use of ivermectin + albendazole 3 (3.0%) reflects occasional incorporation of antiparasitic therapy. Overall, the findings highlight selective use of FDCs, with a clear predominance of cefuroxime-based combinations.

Other drug prescriptions

The other drugs prescribed in the orthopedic department were summarized in Table 3. Among gastroprotective agents, ranitidine, 62 (62.6%), was more frequently prescribed than pantoprazole, 46 (46.5%), indicating substantial use of acid-suppressive therapy. Analgesic use was prominent, with tramadol, 59 (59.6%), showing high utilization, supported by non-steroidal agents such as diclofenac (38.4%) and paracetamol (22.2%), reflecting a multimodal approach to pain management. Among cardiovascular drugs, amlodipine, 19 (19.1%), was more commonly used than nifedipine, 1 (1.0%), suggesting a preference for long-acting antihypertensive therapy. Human Actrapid 17 (17.2%) indicates moderate use of insulin for glycemic control. Nutraceutical supplementation was also notable, particularly trypsin, 59 (59.6%); followed by Calcium + Vitamin D3, 38 (38.4%); and Vitamin K, 6 (6.06%). Overall, the prescribing pattern reflects extensive use of supportive therapies alongside primary treatment, especially for pain management and nutritional supplementation.

**Table 3:** Other drugs prescribed in the orthopedic department. Data have been represented as numbers (percentage).

Class of drugs and percentage	Drugs	N (%)
Antacids	Pantoprazole	46 (46.5)
	Ranitidine	62 (62.6)
Opioids	Tramadol	59 (59.6)
Non-steroidal anti-inflammatory drugs	Paracetamol	22 (22.2)
	Diclofenac	38 (38.4)
Calcium channel blockers	Amlodipine	19 (19.1)
	Nifedipine	1 (1.0)
Human insulin	Human Actrapid	17 (17.2)
Nutraceuticals	Vitamin K	6 (6.06)
	Trypsin	59 (59.6)
	Calcium + Vitamin D3	38 (38.4)

Other FDCs prescriptions

**Figure 2:**
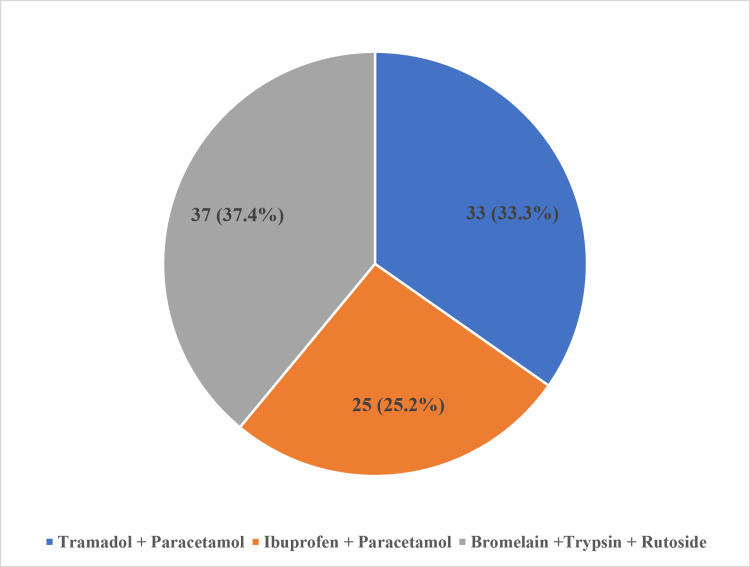
Other FDCs prescribed in the orthopedic department. Data have been expressed as numbers (percentage).

Combination therapy was commonly employed for symptomatic management, with bromelain + trypsin + rutoside being the most frequently prescribed, 37 (37.4%), indicating a strong preference for enzyme-based anti-inflammatory agents. This was followed by tramadol + paracetamol, 33 (33.3%), reflecting reliance on opioid-non-opioid combinations for enhanced pain control. Ibuprofen + paracetamol, 25 (25.2%), also showed considerable use, supporting a multimodal analgesic approach. Overall, the findings suggest predominant use of combination therapies to achieve synergistic effects in pain and inflammation management, as illustrated in Figure 2.

NLEM drug prescriptions

The NLEMs prescribed in the orthopedics department were listed in Table 4. The prescribing pattern in the orthopedics department revealed frequent utilization of analgesics, antimicrobials, and supportive medications listed in the NLEM. Tramadol, 59 (8.2%), was the most commonly prescribed drug, followed by metronidazole, 54 (7.5%); ciprofloxacin, 47 (6.5%); and pantoprazole, 46 (6.4%), reflecting the emphasis on pain management, infection control, and gastrointestinal prophylaxis. Among antibiotics, ceftriaxone, 43 (5.9%), and gentamicin, 17 (2.3%), were commonly used, indicating reliance on broad-spectrum antimicrobial coverage in orthopedic practice. Diclofenac, 38 (5.3%), and paracetamol, 22 (3.0%), were also frequently prescribed for analgesia. Supportive agents such as vitamin C 25 (3.5%) and antihypertensives like amlodipine 19 (2.6%) were prescribed to selected patients with comorbid conditions. Overall, the findings demonstrate predominant use of essential medicines targeting postoperative pain, infection prevention, and supportive care in orthopedic patients.

**Table 4:** NLEMs prescribed in the orthopedics department. Values were expressed as N (%) unless otherwise stated. NLEM: National List of Essential Medicines.

NLEM-prescribed drugs	N (%)
Cefotaxime	2 (0.3)
Cefuroxime	9 (1.2)
Ceftriaxone	43 (5.9)
Clarithromycin	1 (0.1)
Ciprofloxacin	47 (6.5)
Doxycycline	1 (0.1)
Gentamicin	17 (2.3)
Metronidazole	54 (7.5)
Mebendazole	5 (0.7)
Diethylcarbamazine	5 (0.7)
Linezolid	1 (0.1)
Pantoprazole	46 (6.4)
Tramadol	59 (8.2)
Paracetamol	22 (3.0)
Diclofenac	38 (5.3)
Vitamin K	6 (1.0)
Vitamin C	25 (3.5)
Amlodipine	19 (2.6)
Nifedipine	1 (0.1)
Piperacillin + tazobactam	2 (0.3)

WHO core prescribing indicators

Table 5 describes the WHO core prescribing indicators in the orthopedics department. This revealed a relatively high average of 7.29 drugs/encounter. The WHO recommends an average of approximately 1.6-1.8 drugs per prescription as a reference for rational prescribing. In comparison, the study observed a significantly higher average of 7.29 drugs per encounter, indicating a substantial degree of polypharmacy among orthopedic patients. The mean frequency of antibiotics per prescription was 2.37, indicating frequent use of multiple antimicrobial agents, likely related to perioperative prophylaxis and infection management. Injectable formulations were prescribed in 45.4% of prescriptions, reflecting the substantial use of parenteral therapy in acute and postoperative orthopedic care. Additionally, 55.8% of drugs belonged to NLEMs. Overall, the prescribing indicators suggest extensive utilization of multidrug and injectable therapies in orthopedic practice, with a reasonably satisfactory incorporation of essential medicines.

**Table 5:** WHO core prescribing indicators in the orthopedics department. NLEM: National List of Essential Medicine.

Prescribing indicators	Values
Frequency of drugs/encounter (Mean)	7.29
Frequency of antibiotics/prescription (Mean)	2.37
Injectable prescriptions (%)	45.4 %
Drugs belonged to NLEM (%)	55.8 %

## Discussion

A physician’s prescription can be considered a reflection of their perception of the disease and their confidence in pharmacological interventions. It also provides insight into the functioning and characteristics of the healthcare delivery system. Enhancing the overall standards of medical care within this system can contribute to improved quality of life. The incorporation of standard-setting and performance evaluation in clinical practice serves as an effective approach to assess the quality of care [14]. Studies on antibiotic utilization offer a comprehensive overview of current prescribing practices, enabling timely interventions to encourage rational pharmacotherapy [4]. Accordingly, this cross-sectional observational study was undertaken to assess the drug utilization pattern of antimicrobials and additional drugs prescribed in the the orthopedic department at our tertiary care hospital.

Socio-demographic characteristics

In the present study, nearly half of the participants were elderly, with patients aged above 61 years constituting 47.5% of the total sample (n = 99). This observation is in agreement with findings reported by Sini et al., who also noted a predominance of geriatric patients [1]. The next most represented groups were individuals aged 21-40 years (29.3%) and 41-60 years (20.2%). A comparable age distribution was documented by Karki et al., where young- and middle-aged adults formed a substantial proportion of the study population [15]. Gender distribution revealed a higher proportion of males (62.6%) compared to females (37.4%). Similar findings were reported by Abraham et al. This male predominance may be explained by sociocultural factors, as men are more frequently involved in outdoor and occupational activities, increasing their exposure to trauma and related injuries [16].

Falls were recognized as the most common cause of traumatic orthopedic injuries, accounting for 58.6% of cases. This observation is consistent with international studies by Manwana et al., who also reported falls as the leading cause of such injuries [17]. These findings suggest a higher susceptibility of the elderly population to fall-related morbidity, aligning with reports by Alsumadi et al., which highlighted an increased risk of falls among older individuals [18]. Road traffic accidents constituted the second most frequent cause (35.3%), with a higher incidence observed in the 21-40 years age group (54.3%). Similar patterns were reported by Dsouza et al. Since this age group represents the economically productive segment of society, trauma in this population can have significant socioeconomic implications [19].

Antimicrobial drug classes prescriptions

Cephalosporins were the most commonly prescribed class of antimicrobials (54.5%), with ceftriaxone (43.4%) being the predominant agent, followed by cefuroxime (9.1%). Comparable trends have been reported by Pandiamunian et al., who observed predominant use of third-generation cephalosporins in orthopedic settings [20]. Their widespread use may be attributed to broad-spectrum coverage, convenient dosing, favorable safety profile, and efficacy against diverse microorganisms. Metronidazole, a 5-nitroimidazole derivative effective against anaerobic organisms, was prescribed in 54.5% of cases. Ciprofloxacin (47.5%), a broad-spectrum fluoroquinolone active mainly against Gram-negative bacteria with moderate Gram-positive coverage, and aminoglycosides such as gentamicin (17.2%) were also frequently utilized. Similar prescribing patterns were reported by Shankar et al., who documented extensive use of these agents in hospitalized patients [21]. Overall, the sequence of antimicrobial use, cephalosporins followed by nitroimidazoles, fluoroquinolones, aminoglycosides, anthelminthics (10.1%), tetracyclines (3.0%), and macrolides (1.0%), corresponds with findings by Beg et al., indicating consistency in prescribing practices across tertiary care centers [22].

The preference for second-generation cephalosporins and β-lactam antibiotics combined with β-lactamase inhibitors has increased, as older agents and monotherapy may be less effective. In the present study, the FDC of cefuroxime with sulbactam (25.2%) was commonly prescribed. Such combinations provide broader antimicrobial coverage and improved efficacy against both Gram-positive and Gram-negative organisms. The β-lactamase inhibitor protects the β-lactam ring from enzymatic degradation, thereby enhancing therapeutic effectiveness. Similar findings were reported by Sinha et al., who emphasized the clinical benefits of β-lactam/β-lactamase inhibitor combinations [23]. The combination of piperacillin with tazobactam accounted for 2.0% of prescriptions, consistent with observations by Beg et al., particularly in cases requiring extended Gram-negative coverage [22].

The use of gastroprotective drugs was also quite common, with ranitidine (62.6%) and pantoprazole (46.5%) being the most prescribed drugs in this category. It is important to note that this can be explained by the fact that the administration of acid-lowering medications, together with NSAIDs and opioids, is common practice in order to reduce the negative impact on the stomach. The results obtained from the research agree with the study conducted by Tsumura et al., which indicated that H2 receptor antagonists and proton pump inhibitors are useful for preventing NSAID ulcers [24]. With regard to painkillers, the use of tramadol was most common (59.6%), followed by diclofenac (38.4%) and paracetamol (22.2%). These results are comparable to those reported by Sachdev et al., reflecting the necessity for effective pain management in orthopedic and postoperative patients [25].

Among patients with cardiovascular comorbidities, antihypertensive medications, particularly calcium channel blockers such as amlodipine and nifedipine, were commonly used for blood pressure control. Similar trends have been noted by Mutharasan et al., who reported frequent use of calcium channel blockers in hospitalized patients [26]. Additionally, supportive therapies including trypsin (59.6%), calcium with vitamin D3 (38.4%), and vitamin K (25.2%) were widely prescribed. These observations are consistent with Nagla et al., who reported similar use of nutritional and enzyme supplements to facilitate recovery in orthopedic patients [27].

Prescription of FDCs, other FDCs

Among the other FDCs, tramadol with paracetamol (33.3%) was prescribed more frequently than ibuprofen with paracetamol (25.2%). This pattern aligns with findings reported by Nagla et al., suggesting a preference for opioid-non-opioid combinations to achieve superior analgesic effects [27]. Additionally, the combination of bromelain, trypsin, and rutoside (37.4%) was commonly utilized, likely due to its role in reducing inflammation and edema and promoting postoperative healing.

Prescription of NLEM drugs

In the present study, 55.8% of the prescribed antimicrobial agents belonged to the National Essential Drug List. Similar findings were reported by Abraham et al. and Muraraiah et al., who observed a comparable proportion of prescriptions from the essential medicines list in their respective drug utilization studies. This similarity indicates a moderate level of compliance with essential medicine prescribing guidelines [16,28].

WHO core prescribing indicators

The mean frequency of drugs prescribed/encounter was 7.29, indicating a relatively high prevalence of polypharmacy among the study population. This observation is in agreement with earlier reports in the literature, where the mean frequency of drugs/prescription ranged between 5.1 and 12 [29]. Multiple antimicrobial agents were commonly prescribed to individual patients, as evidenced by an average of 2.37 antibiotics per prescription. Injectable medications were also used extensively, with 45.4% of prescriptions containing parenteral formulations. In addition, 55.8% of the prescribed drugs were from the NLEM.

Limitations

Certain limitations of this study should be acknowledged. The study was conducted in a single tertiary care hospital, which may restrict the generalizability of the findings to other settings. The cross-sectional design limits the ability to establish causal relationships between prescribing patterns and clinical outcomes. Furthermore, the sample size may not fully represent the broader orthopedic patient population. Conducting multicenter studies would provide a more comprehensive evaluation.

## Conclusions

The present study was conducted to evaluate the pattern of antimicrobial utilization among patients admitted to the orthopedic department of a tertiary care hospital. The analysis included assessment of age-wise and gender-wise distribution, class of antimicrobials prescribed, use of FDCs, and adherence to the essential drug list. The majority of patients belonged to the adult (21-40 years) and elderly (>61 years) age groups, indicating higher orthopedic admissions in the economically productive and geriatric populations. Antimicrobial utilization was higher among male patients, which corresponded with the overall male predominance in the study population. Among cephalosporins, ceftriaxone was the most frequently prescribed agent across age groups, reflecting its broad-spectrum coverage and common empirical use in orthopedic infections. Cefuroxime and its FDCs were also widely utilized. Fluoroquinolone (ciprofloxacin) use was notable, particularly in elderly patients, likely due to its good bone penetration and role in managing Gram-negative infections. Aminoglycosides such as gentamicin and tobramycin were prescribed mainly in adult males, possibly reflecting their use in severe infections and postoperative cases. Metronidazole was commonly prescribed, particularly in adult and elderly populations, suggesting management of mixed or anaerobic infections. More than half of the drugs were prescribed from the Essential Drug List, reflecting good adherence to rational prescribing principles, with further opportunities to strengthen and optimize prescribing practices.
